# Screening for Cognitive Function in Primary Care

**DOI:** 10.1093/geroni/igaa057.1947

**Published:** 2020-12-16

**Authors:** Tara Cortes, Liz Seidel

**Affiliations:** 1 New York University, New York, New York, United States; 2 NYU Rory Meyers College of Nursing, New York, New York, United States

## Abstract

A comprehensive program for early detection and treatment of Alzheimer’s and related dementias requires a systematic process for provider education, assessment of cognitive function and referral to appropriate resources when indicated. This discussion will focus on the implementation of cognitive screening into primary care. Over 200 staff across 11 primary care sites were trained on screening for dementia in the annual wellness visit. In the following 6 months nearly 2000 annual wellness visits were conducted. The annual wellness visit was designed to include a cognitive screen. 83% of the patients were asked the cognitive screen questions and 13% responded “yes” to the question. Further testing was offered to that group and 71% of those who had responded “yes” agreed to be tested with a picture memory screen. 32% scored less than 5 on that screen and were referred to gero-psychiatry, geriatricians and community based organizations for caregiver support services.

